# Investigating gene expression profiles of whole blood and peripheral blood mononuclear cells using multiple collection and processing methods

**DOI:** 10.1371/journal.pone.0225137

**Published:** 2019-12-06

**Authors:** Aarti Gautam, Duncan Donohue, Allison Hoke, Stacy Ann Miller, Seshamalini Srinivasan, Bintu Sowe, Leanne Detwiler, Jesse Lynch, Michael Levangie, Rasha Hammamieh, Marti Jett

**Affiliations:** 1 US Army Center for Environmental Health Research, Fort Detrick, MD, United States of America; 2 The Geneva Foundation, US Army Center for Environmental Health Research, Fort Detrick, MD, United States of America; 3 Oak Ridge Institute for Science and Education, Fort Detrick, US Army Center for Environmental Health Research, Fort Detrick, MD, United States of America; The Ohio State University, UNITED STATES

## Abstract

Gene expression profiling using blood samples is a valuable tool for biomarker discovery in clinical studies. Different whole blood RNA collection and processing methods are highly variable and might confound comparisons of results across studies. The main aim of the current study is to compare how blood storage, extraction methodologies, and the blood components themselves may influence gene expression profiling. Whole blood and peripheral blood mononuclear cell (PBMC) samples were collected in triplicate from five healthy donors. Whole blood was collected in RNAgard^®^ and PAXgene^®^ Blood RNA Tubes, as well as in collection tubes with anticoagulants such as dipotassium ethylenediaminetetraacetic acid (K_2_EDTA) and Acid Citrate Dextrose Solution A (ACD-A). PBMCs were separated using sodium citrate Cell Preparation Tubes (CPT^™^), FICOLL^™^, magnetic separation, and the LeukoLOCK^™^ methods. After blood collection, the LeukoLOCK^™^, K_2_EDTA and ACD-A blood tubes were shipped overnight using cold conditions and samples from the rest of the collection were immediately frozen with or without pre-processing. The RNA was isolated from whole blood and PBMCs using a total of 10 different experimental conditions employing several widely utilized RNA isolation methods. The RNA quality was assessed by RNA Integrity Number (RIN), which showed that all PBMC procedures had the highest RIN values when blood was stabilized in TRIzol^®^ Reagent before RNA extraction. Initial data analysis showed that human blood stored and shipped at 4°C overnight performed equally well when checked for quality using RNA integrity number when compared to frozen stabilized blood. Comparisons within and across donor/method replicates showed signal-to-noise patterns which were not captured by RIN value alone. Pathway analysis using the top 1000 false discovery rate (FDR) corrected differentially expressed genes (DEGs) showed frozen vs. cold shipping conditions greatly impacted gene expression patterns in whole blood. However, the top 1000 FDR corrected DEGs from PBMCs preserved after frozen vs. cold shipping conditions (LeukoLOCK^™^ preserved in RNA*later*^®^) revealed no significantly affected pathways. Our results provide novel insight into how RNA isolation, various storage, handling, and processing methodologies can influence RNA quality and apparent gene expression using blood samples. Careful consideration is necessary to avoid bias resulting from downstream processing. Better characterization of the effects of collection method idiosyncrasies will facilitate further research in understanding the effect of gene expression variability in human sample types.

## Introduction

Human blood and peripheral blood mononuclear cells (PBMCs) are critical biological specimen types collected in clinical trials as well as in basic science research. These specimens have been widely used to determine gene expression signatures that may be associated with disease predictions [1–3]. Whole blood is a valuable resource that is readily accessible, whereas PBMCs separation from whole blood is labor intensive and requires several methodological steps that must be strictly followed. Each of these blood-derived RNA sources are known to have inherent characteristics that ultimately result in a unique gene expression profile. It is imperative to detect the transcriptomic changes that can be rapidly translated into clinical practice, and are feasible, reproducible, cost-effective, and easy to implement. Accurate analyses are often complicated by changes caused by sample collection, handling, storage, and extraction methodologies [4–16]. As a general principle, blood samples need to be processed for PBMC separation as soon as possible to preserve the *in vivo* state of the cells. While the standardization of blood sample handling and processing procedures is essential for better comparisons of gene expression results across experiments, very few studies have investigated the influence of sample collection methodology and its impact on whole blood transcriptome analysis. Current literature emphasizes the importance of RNA extraction methods [17, 18] and the same level of standardization and consistency must be applied to the pre-analytical sample collection stage [12, 13, 19].

Multiple commercial kits [4, 20] are available for whole blood RNA collection and immediate stabilization. These kits use proprietary reagents which lyse blood cells and can stabilize nucleic acids immediately upon collection without the need to extract the blood leukocyte component. The samples derived from whole blood capture RNA profiles of all cell types in whole blood including erythrocytes, leukocytes and platelets, whereas the PBMC samples are largely devoid of granulocytes, platelets, and reticulocytes [21].

Although a growing number of published works on gene expression analysis have made comparisons of the RNA sources, few of these reports have compared multiple whole blood and PBMC collection methods [22] and previously published data show significant overlaps between whole blood and PBMC gene expression [21, 23]. The expression profile using PBMCs can be variable and is dependent not only on isolation method, but also on the blood storage conditions [8, 24]. Furthermore, disease can have a significant impact on blood cell type proportions in peripheral blood [25, 26].

Whole blood is considered to be the matrix of choice for its ease of implementation [12], whereas PBMC separation is labor intensive and can introduce additional handling bias to clinical samples [21]. Therefore, the current study aims to test gene expression patterns for whole blood collected in multiple types of sample collection tubes, either preserved immediately or shipped overnight under refrigeration. PBMC separation was also tested using routine centrifugation procedures in addition to recently introduced procedures such as magnetic separation [27] and the LeukoLOCK^™^ method [18].

The primary goal of the current study is to characterize the impact of sample processing and RNA isolation on the observed transcriptional profile of blood cells from healthy donors using the Agilent-array platform. We have used a balanced experimental design to eliminate certain statistical biases in the analysis, permitting us to effectively assess gene expression profiles using comparisons of multiple blood sampling techniques, whole blood vs. PBMCs, and shipping temperatures, as well as RNA extraction methods. Consequently, if gene expression needs to be analyzed, it would be necessary to consider the variability of methodologies in the processing of whole blood and PBMCs.

## Materials and methods

### Sample collection

**Fig 1:**
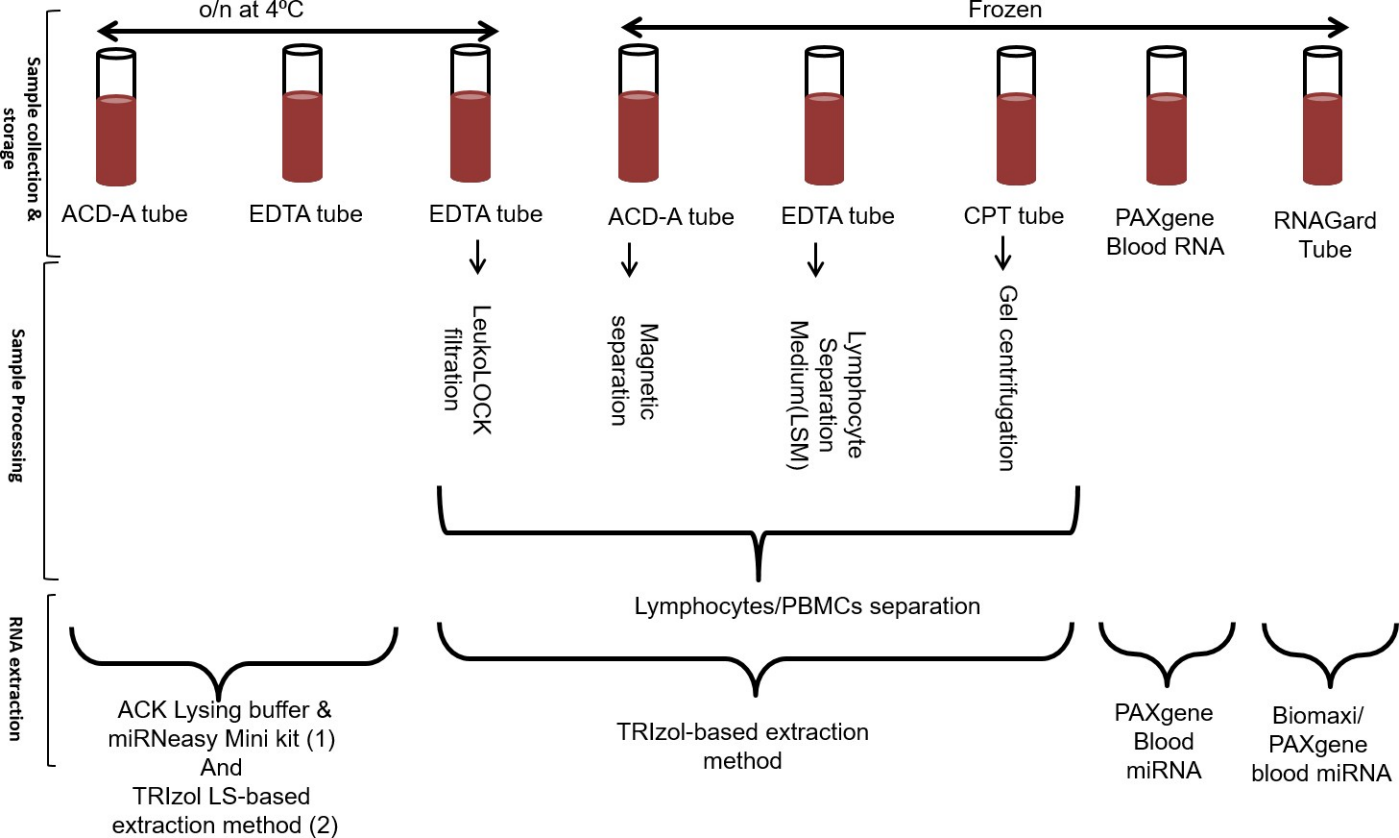
Sample collection, processing and RNA extraction: Whole blood was collected in triplicate followed by peripheral blood mononuclear cells (PBMCs) separation in a subset of samples. The following sample collection tubes were used for the study: RNAgard^®^, PAXgene^®^ RNA, EDTA, ACD-A, and CPT tubes. The PBMC separation was done using standard procedures for CPT tubes, and magnetic bead, LeukoLOCK^™^ and LSM methods. The samples were then stored at either 4°C (Cold) or -80°C (Frozen) and shipped overnight (o/n) for follow-up RNA extraction. Next, they were treated with one or more of several different RNA extraction procedures: Biomaxi Precip Buffer/ PAXgene^®^ Blood miRNA, PAXgene^®^ Blood miRNA, TRIzol^®^ LS, ACK Lysing Buffer/ Qiagen miRNeasy, and TRIzol^®^ Reagent manufacturer’s protocol.

Research not involving human subjects determination was made at USACEHR as blood was purchased from a commercial supplier. Whole blood was bought and collected at the AllCells, LLC facility (Alameda, CA, USA) from five different donors as shown in Fig 1. The blood collection from the five volunteers was carried out on two subsequent days with collection of two volunteers’ samples the first day and three volunteers’ samples the second day. For each donor, the blood was collected in triplicate directly into PAXgene^®^ blood RNA tubes (PreAnalytix, QIAGEN, Inc., Germantown, MD, USA) (2.5 mL whole blood + 6.9 mL of PAXgene^®^ RNA solution), RNAgard^®^ blood tubes (Biomatrica, Inc., San Diego, CA, USA) (2.5 mL of whole blood + 6.65 mL of RNAgard^®^ preservative), into three separate sets of BD Vacutainer^™^ K_2_EDTA (BD, Franklin Lakes, NJ, USA) spray-coated collection tubes with 3 mL of whole blood per tube, BD Vacutainer^™^ CPT tubes (BD) with 8 mL of whole blood collected per tube, and Acid Citrate Dextrose Solution A (ACD-A) tubes (BD) (8.5 mL whole blood + 1.5 mL of ACD-A). The RNA was stabilized in PAXgene^®^ blood RNA, RNAgard^®^, EDTA, ACD-A, and CPT tubes by slowly inverting the tubes eight times after collection. After stabilization, the RNAgard^®^ tubes and the PAXgene^®^ blood RNA tubes were immediately frozen, one set of triplicate EDTA tubes was stored at 4°C, and the remaining tubes received further processing at the AllCells, LLC facility as described.

### BD vacutainer^™^ CPT tube procedure

For the BD Vacutainer^™^ CPT Tubes, 8 mL of whole blood was collected into the tube containing 1.0 mL of 0.1 M Sodium Citrate Solution (Top Fluid Layer), 3.0 gm of Polyester Gel (Middle Layer), and 2.0 mL of Polysaccharide/Sodium Diatrizoate Solution (FICOLL^™^ Hypaque^™^ solution, Bottom Fluid Layer). Immediately following blood collection, the tubes were inverted and centrifuged following manufacture’s guidelines to allow for the separation of the blood components. After centrifugation, 3 mL of plasma was removed from the uppermost layer. The PBMC layer was gently suspended in the remaining plasma and transferred into 15-mL conical tubes and washed twice with 5 mL of 1x DPBS (Corning, Inc., Corning, NY, USA) by centrifugation at 300 × *g* for 10 min. The PBMC cell pellet was resuspended in 0.5 mL of TRIzol^®^ Reagent (Invitrogen, Life Technologies, Grand Island, NY, USA) and immediately frozen.

### ACD-A tubes and the magnetic bead-based separation method for PBMCs

Immediately following blood collection and tube inversion, the ACD-A tube had 1.5 mL of whole blood removed for the magnetic bead separation method, and the remaining whole blood was saved at 4°C for future processing. The 1.5 mL aliquot was processed through the EasySep^™^ Direct Human Total Lymphocyte Isolation Kit (STEMCELL Technologies, Inc. Cambridge, MA, USA) for negative selection, according to the manufacturer’s procedure. Briefly, the magnetic beads and an isolation cocktail were added to the whole blood and the sample was incubated on the EasySep^TM^ Magnet and transferred to a new tube. Again, magnetic beads were added and the sample was incubated on the EasySep^TM^ Magnet, and the enriched cell suspension was then placed in a new tube. After isolation, the sample was centrifuged and pelleted, washed with 1x DPBS, resuspended in 0.5 mL of TRIzol^®^ Reagent, and immediately frozen.

### EDTA tube and the Lymphocyte Separation Medium

One set of the triplicate EDTA blood tubes from each donor was used to isolate PBMCs with Lymphocyte Separation Medium (LSM) (Corning, Inc.), according to the manufacturer’s procedure. Briefly, immediately after blood collection and tube inversion, 3 mL of LSM was added to a 15 mL tube and carefully overlaid with 3 mL of whole blood from the EDTA tube and centrifuged 400 x *g* for 30 min at room temperature. The PBMC interface was carefully removed by pipetting and was washed twice with 5 mL 1x of DPBS with centrifugation at 250 × *g* for 10 min. PBMC pellets were resuspended in TRIzol^®^ Reagent (Invitrogen) and immediately frozen.

### EDTA blood tube and LeukoLOCK^™^ processing

One set of the triplicate EDTA blood tubes from each donor was processed through the LeukoLOCK^™^ Total RNA Isolation System (Life Technologies, Grand Island, NY, USA) according the manufacturer’s procedure. Briefly, immediately after blood collection and tube inversion, 3 mL of blood was drawn through the LeukoLOCK^™^ filter, washed with 1xPBS (Life Technologies, Grand Island, NY, USA), saturated with RNA*later^®^* (Life Technologies, Grand Island, NY, USA), the inlet ports of the filter were sealed, and the filter was stored at 4°C.

### Shipping and storage conditions

After blood collection and initial processing at the AllCells, LLC facility, the whole blood in RNAgard^®^ and PAXgene^®^ RNA Blood tubes, and the PBMCs in TRIzol^®^ Reagent from the whole blood in the EDTA, ACD-A, and CPT tubes were shipped overnight to our facility on dry ice and stored at– 80°C until extraction. The whole blood collected in the EDTA and the ACD-A tubes and the LeukoLOCK^™^ filters were shipped at 4°C for overnight delivery. The whole blood collected in the EDTA and ACD-A tubes was processed immediately for extraction upon arrival, and the RNA*later*-stabilized PBMCs collected in the LeukoLOCK^™^ filter was stored at 4°C until processing. Due to the samples being collected and shipped on two subsequent days, the sample extractions that were processed immediately upon arrival were performed on two different days. To minimize batch effects, the identical extractions were performed by the same technician on both days. For the samples that arrived frozen or the LeukoLOCK^™^ filters that were stabilized in RNA*later*, we waited until we had the samples from all five donors so that they could all be processed together on the same day by the same technician which caused the samples to be stored for different numbers of days prior to processing.

### RNA extraction methods

#### TRIzol^®^ LS extraction of PBMCs

TRIzol^®^ LS Reagent RNA extraction was conducted on whole blood from EDTA and ACD-A tubes immediately upon arrival according to the manufacturer’s protocol with a few minor modifications during the initial steps. Briefly, the original blood tube was inverted 3–5 times and 1 mL of whole blood was aliquoted into a 15 mL conical tube, 1 mL of nuclease-free water was added, and the sample was inverted. Next, 6 mL of TRIzol^®^ LS Reagent was added, the sample was incubated at room temperature for 5 min before chloroform (Sigma-Aldrich, St. Louis, MO, USA) was added, and the sample was inverted and incubated for 3 min at room temperature. This was followed by a 15-minute centrifugation at 12,000 x *g* at 4°C. The upper aqueous phase containing the RNA was transferred to a new tube. Isopropanol (Sigma-Aldrich, St. Louis, MO, USA) was added and the sample was incubated for 10 min at room temperature, followed by a 12,000 x *g* centrifugation at 4 ᴼC for another 10 min. The RNA pellet was washed with 75% ethanol (Sigma-Aldrich) and air dried for 10 min before being resuspended in 30 μL of RNase-free water (Ambion, ThermoFischer, Scientific, Inc.).

#### TRIzol^®^ extraction of PBMCs

The PBMCs isolated from the EDTA, CPT, and ACD-A tubes were received and then kept at -80°C for 1 or 2 days and extracted using TRIzol^®^ Reagent according to the manufacturer’s procedure for RNA extraction.

#### TRIzol^®^ extraction from LeukoLOCK^™^ filter

The LeukoLOCK^™^ filters were stored at 4°C for 2 or 3 days prior to processing according to the manufacture’s protocol. Briefly, the RNA*Later^®^* was removed from the LeukoLOCK^™^ filter, and the filter was flushed with 3 mL of TRIzol^®^ Reagent and the flow-through was collected. For extraction, 1 mL of the collected TRIzol^®^ Reagent was then used for PBMC extraction following the manufacturer’s procedure for TRIzol^®^ extraction of RNA.

#### PAXgene^®^ blood RNA tubes: PAXgene^®^ blood miRNA kit

Whole blood collected in PAXgene^®^ Blood RNA tubes was stored at -80°C for 1 to 2 days after being received, and then was thawed and incubated overnight at room temperature to ensure complete lysis of blood cells and maximize the mRNA yield. The PAXgene^®^ Blood RNA tubes were handled following our basic laboratory protocol based off of the PreAnalytiX specimen handling and enhanced yield procedures from PAXgene Blood RNA MDx Kit Handbook (08/2016). Samples were processed using the PAXgene^®^ Blood miRNA Kit (PreAnalytix, Inc., Qiagen, Germantown, MD, USA) following the manufacturer’s automated QIAcube (Qiagen, Germantown, MD, USA) protocol. Briefly, the PAXgene^®^ Blood RNA tubes were centrifuged for 10 min at 3500 x *g*. The supernatant was discarded and the pellet was washed with RNase-free water, the tube was vortexed to thoroughly to resuspend the pellet, followed by another centrifugation for 10 min at 3500 x *g*. The supernatant was discarded and the pellet was resuspended in 350 μL of Buffer BM1 (Qiagen, Germantown, MD, USA). The sample was vortexed until the pellet was visibly dissolved, and the sample was transferred into a 2 mL processing tube and loaded into the QIAcube (Qiagen, Germantown, MD, USA) along with the other required reagents per manufacturer’s instructions. Briefly, the automated RNA purification protocol consists of 2 parts, “PAXgene Blood miRNA Part A” in which the QIAcube performs the steps of the protocol through to elution of RNA, and “PAXgene Blood miRNA Part B” where heat denaturation of samples at 65°C is performed by the QIAcube.

#### ACK Lysing Buffer/ miRNeasy protocol

RNA was extracted from 2 mL of whole blood from EDTA blood tubes and from 6 mL of whole blood from ACD-A blood tubes using ACK Lysing Buffer (Lonza, Inc., Walkersville, MD, USA) followed by the miRNeasy Mini Kit (Qiagen, Germantown, MD, USA). The original whole blood tubes were stored and shipped at 4°C after blood collection, and immediately processed upon being received by inverting 3–5 times and the whole blood aliquot was spun down at 400 x *g* for 10 min at 4°C. The supernatant was discarded and an equivalent volume of ACK Lysing Buffer was added to the remaining pellet, which was gently resuspended by swirling the tube for 30–60 seconds. The pellet was washed twice using 1x PBS (Lonza, Inc., Basel, Switzerland) by centrifuging at 400 x *g* for 10 min at 4°C. The pellet then underwent procedures specified by the miRNeasy Mini Kit protocol. Briefly, the pellet was lysed with QIAzol ^®^Lysing Reagent (Qiagen, Germantown, MD, USA) and phase separation was then induced by centrifuging the samples with chloroform. The aqueous phase was removed, ethanol was added, and the sample was placed in the RNeasy Mini column. Columns were centrifuged with Buffer RWT and Buffer RPE to purify the RNA, which was then eluted in 50 μL of Nuclease-free water.

#### RNAgard^®^ blood tubes: BioMaxi^™^ precipitation/ PAXgene^®^ blood miRNA protocol

The RNAgard^®^ tubes were stored at -80°C for 1 to 2 days after being received, and then thawed and incubated overnight at room temperature. The tube was inverted 3–5 times, and 1.33 mL of BioMaxi^™^ Precipitation Buffer (Biomatrica, CA, USA) was added to a 4 mL aliquot of the RNAgard^®^ whole blood. The sample was mixed vigorously, incubated at room temperature for 15 min, centrifuged at 4,500 x *g* for 30 min at room temperature in a swinging bucket rotor, and the supernatant was discarded. The pellet was then resuspended in 350 μL of Buffer BM1 (Qiagen, Germantown, MD, USA), and processed in the QIAcube (Qiagen, Germantown, MD, USA) according to the procedure for PAXgene^®^ Blood miRNA.

### RNA quality and quantification

Following isolation, the RNA concentration was measured using a NanoDrop ND-2000 spectrophotometer (NanoDrop Technologies, Wilmington, DE, USA). The quality of the RNA was evaluated on a TapeStation System (Agilent Technologies, Inc., Santa Clara, CA, USA) to get the RIN value.

### Gene expression array

The microarray was carried out on Agilent Technologies Human Gene Expression Arrays (039494) using a Quick Amp Low Input Labeling kit and Agilent RNA Spike-In Two-Color kit following the Two-Color Microarray-based Gene Expression Protocol by Agilent Technologies. Briefly, experimental RNA (200 ng) was labeled with Cy-5 dye and co-hybridized with Cy-3 dye labeled human reference RNA (Human Universal Reference RNA, Agilent). Samples were fragmented and hybridized at 65°C for 17 hours and were subsequently washed according to the Agilent Gene Expression Hybridization protocol. Slides were scanned on the SureScan Microarray Agilent Scanner System. Feature Extraction software (version 12.0.3.1) provided the extracted features for data analysis.

### Data analysis

Chips were read with Agilent Feature Extraction software and all subsequent analysis was performed using custom R scripts. Microarray data was analyzed using limma package in the R programming language [28]. Agilent feature extraction files were read using the recommended agilent.median option of the limma “read.maimages” function. The resulting median intensity values for the red and green channels were then normalized using the within array “loess” method in which locally weighted regression was applied to align the two color channels. The output of this step is a normalized probe intensities (A values) and a log2 ratio of normalized red to green channel intensities (M values). Next, the resulting M values were between array normalized using the”quantile” option of the limma normalize BetweenArrays function. This brings the array to array distributions of M values into close alignment. Duplicate probe M values were then averaged to create a single normalized intensity log2 ratio value for each distinct probe on the arrays. Determination of significant probe differences between arrays was made using the lmFit function of limma in which a probe-wise linear model is fit to generate regression coefficients and associated empirical Bayes moderated t-statistics and p-values. Benjamini and Hochberg false discovery rate correction (FDR) was used to control for type 1 family-wise error rate. Differentially expressed genes (DEGs) were designated as those having probes with FDR corrected p-values 0.05 unless otherwise noted. Other common normalization methods were investigated and their use did not change the general findings. Likewise, enforcing a 50% probe variance cutoff filter to highlight technical variation produced very similar results. Manhattan distances were calculated and were plotted using Sammon plot analysis [29]. Pathway analysis was carried out using Ingenuity Pathway Analysis (IPA) (Qiagen, Germantown, MD USA, Inc.). The data from this study was also submitted to GEO with # GSE113395.

### Network analysis

The Top 1000 DEGs (based on the p values) obtained from comparing whole blood at cold vs the frozen conditions were uploaded into Ingenuity Pathway Analysis software (Ingenuity Systems, Redwood City, CA) and mapped to the functional networks available in the Ingenuity Pathway Knowledge Base. The expression analysis using IPA was done using default settings and stringent filter for tissues and primary cells settings. The top physiological system and biofunctions of the core analysis was conducted using the default settings for our dataset. P-values were corrected for multiple testing using the Benjamini-Hochberg (B-H) false discovery rate determining the association between the genes in the dataset and the biofunctions these are associated.

## Results and discussion

### Collection tubes to determine gene expression changes

The objective of this study was to investigate how blood storage, extraction methodologies, and the blood component itself may influence what genes appear to be expressed in downstream applications. To determine the gene expression signatures, whole blood was collected in triplicate from five healthy volunteers and analyzed (Fig 1). The whole blood was collected in commonly used anticoagulant tubes such as K_2_EDTA and ACD-A as it has been previously shown that platelet counts in citrated blood samples like those collected in the ACD-A tubes are lower than those in EDTA samples [30]. The PAXgene^®^ RNA and RNAgard^®^ blood tubes integrate the key steps of whole blood collection and intracellular RNA stabilization and have been referred to as the standard collection methods for RNA–based studies [31]. We used standard procedures for PBMCs separation, such as FICOLL^™^, CPT tubes, magnetic separation, and the LeukoLOCK^™^ method. The FICOLL^™^ method uses density differences between mononuclear cells and other elements in blood fluid, whereas CPT tubes simplifies this procedure using a blood collection tube containing a citrate anticoagulant with a FICOLL^™^ density fluid and a polyester gel barrier. CPT and FICOLL^™^ PBMC isolation protocols have the same ability to purify high quality immune cell subpopulations as indicated by no difference in the gene expression profiles between the immune cells obtained by these two methods [7]. The magnetic separation procedure facilitates 99.9% red blood cell (RBC) depletion without the need for density gradient centrifugation, making it more time efficient. Isolated PBMCs must be preserved immediately for future RNA extraction. LeukoLOCK^™^ is a filter-based method that retains leukocytes on top of the filter and depletes erythrocytes. Once the leukocytes are lysed on the filter, they can be preserved at room temperature or 4°C and can be used as the method of choice in non-clinical setting [32]. All the RNA extractions were done using routine procedures and since it is reported that lysis reagents may produce artifacts when used to isolate leukocytes [33], we performed the RNA extraction from whole blood with and without RBC lysis. After extractions were completed, the samples were quantified using spectrophotometry, and quality assessed using the RNA integrity number (RIN) value on the Agilent TapeStation.

### RNA quality and quantity

The RNA quantity per milliliter of blood (Table 1) was calculated and RNA isolated from RNAgard^®^ Blood Tubes was higher (mean with standard deviation 3.7 μg ±1.5) than PAXgene^®^ Blood Tubes (1.7 μg ± 0.44). The whole blood without RBC lysis had a much higher RNA concentration from EDTA (18.35 μg ± 10.17) as well as ACD-A tubes (31.07 μg ±20.98) when compared to RBC lysis using ACK Lysing Buffer from EDTA (0.86 μg ± 0.89) and ACD tubes (0.24 μg ± 0.18). Comparable results were obtained using PBMC extraction methods where FICOLL^™^ separation methods yielded highest quantity of RNA (5.9 μg ± 2.2) followed by CPT tubes (2.4 μg ± 1.6) and magnetic separation (2.3 μg ± 0.80), and lowest RNA yield using LeukoLOCK^™^ filtration device (1.1 μg ± 0.79). It has been observed that PAXgene^®^ RNA and Tempus Blood RNA tubes produce high quality RNA with sufficient yield [20] and a consistent expression profile [34]. We did not include Tempus Blood RNA tubes in our study but observed that all methods tested yielded sufficient material for subsequent assays. LeukoLOCK^™^ has shown lowest yield but high RNA integrity along with low DNA contamination when tested previously [18]. Also, LeukoLOCK^™^ sample preparation produced the best quality and yield of RNA when TRIzol^®^ Reagent with lysis buffer and Tempus Blood RNA tubes were used with equine blood [17]. Additionally, it has been reported that blood samples frozen in TRIzol^®^ Reagent after RBC removal effectively preserved RNA quality and that extractions with TRIzol^®^ Reagent yielded significantly better RNA integrity than extractions without TRIzol^®^ Reagent [35].

**Table 1:** General statistics for RNA concentration (in micrograms) and RNA integrity number (RIN) data per mL of blood. The sample collection tube followed by RNA extraction procedure is listed in the rows. The PBMC separation procedure is also referred as PBMC prep. CV refers to coefficient of variation and SD refers to standard deviation and μg refers to microgram.

Sample	Whole Blood						PBMC			
Tube	EDTA		ACD-A		RNAgard^®^	PAXgene^®^	EDTA		CPT	ACD-A
RNA Extraction	TRIzol^®^ LS	ACK_miRNA	TRIzol^®^ LS	ACKmi RNA	Biomaxi^™^_PAXmiRNA	PAXmiRNA	TRIzol			
PBMC Prep							LeukoLOCK^®^	LSM	CPT	Magnetic
**Mean yield** **(**μ**g)**	**18.4**	**0.9**	**31.1**	**0.2**	**3.7**	**1.7**	**1.1**	**5.9**	**2.4**	**2.3**
**Standard deviation**	10.2	0.9	21.0	0.2	1.5	0.4	0.8	2.2	1.6	0.8
**Range**	24.7	2.0	54.1	0.4	4.0	1.0	2.0	5.3	4.2	2.0
**CV**	55.4	104.1	67.5	74.9	38.9	26.0	73.0	36.6	65.8	35.0
**Mean RIN**	**5.6**	**7.6**	**5.1**	**7.8**	**4.9**	**8.2**	**8.2**	**8.8**	**8.4**	**8.8**
**RIN SD**	1.3	1.7	1.4	1.3	0.9	0.4	0.4	0.3	1.4	0.3

The RNA quality was assessed by RIN value (Table 1) and we found that all PBMC procedures, whether stored cold or stabilized in TRIzol^®^ Reagent, had the highest RIN values with a mean of at least 8.2 ± 0.37 standard deviation. For whole blood extractions, the RNA extracted from the PAXgene^®^ Blood RNA tubes had the highest RIN values (8.2 ± 0.36), with the second highest being whole blood processed using ACK Lysing Buffer (7.6 ± 1.65, EDTA and 7.8 ± 1.27, ACD-A tubes). Table 2 shows the relative signal-to-noise (S/N) ratio averaged for technical repeats within and across individual expression correlations using the different collection systems. We observed that comparisons within and across donor/method replicates showed S/N patterns which were not captured by RIN value alone. The PBMC methods have high RIN values but have low correlation ratios as well as distance ratios. Initial data analysis showed that human blood stored at 4°C overnight performed equally well when compared to frozen stabilized blood based on correlation ratios as well as distance ratios.

**Table 2:** Overview of correlation ratio, distance ratio, and RIN medians. Red denotes more favorable values (high RIN; low distance ratio; high correlation ratio) while green denotes less favorable values. The sample type refers to whole blood and PBMCs and method explains the RNA isolation procedure and/or PBMCs separation procedure.

Sample type	Method	Corr. Ratio	Dist. Ratio	RIN median
PBMC	Magnetic separation	1.024	0.901	8.9
PBMC	CPT tubes	1.024	0.891	9.3
PBMC	LSM	1.019	0.903	9.0
Whole blood	ACD_RBC_lysis	1.092	0.722	8.3
Whole blood	ACD_TRIzol	1.102	0.788	5.6
Whole blood	EDTA_RBC_lysis	1.082	0.827	8.4
Whole blood	ACD_TRIzol	1.075	0.843	5.9
PBMC	LeukoLOCK^™^ separation	1.034	0.909	8.2
Whole blood	PAXgene^®^	1.022	0.885	8.2
Whole blood	RNAgard^®^	1.040	0.801	4.9

### Gene expression data

**Fig 2:**
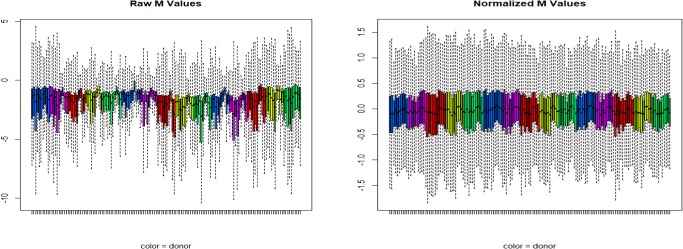
Microarray signal intensity distribution before and after normalization. Raw (a) and normalized (b) microarray probe M values (log2 fold differences) obtained for five different individuals using all different blood collection systems. Colors represent individual donors.

We observed gene expression patterns and Fig 2 shows raw, as well as within-array normalized mRNA expression levels across five different individuals using all different collection methods. The patterns of expression fold values appear mostly consistent across all the collection methods. We observed that PBMC samples differed qualitatively from the whole blood based collection methods and technical repeat samples from individuals tended to cluster together; overall, the profile differences observed between methods were relatively greater than the differences between individual subjects within the methods used.

**Fig 3:**
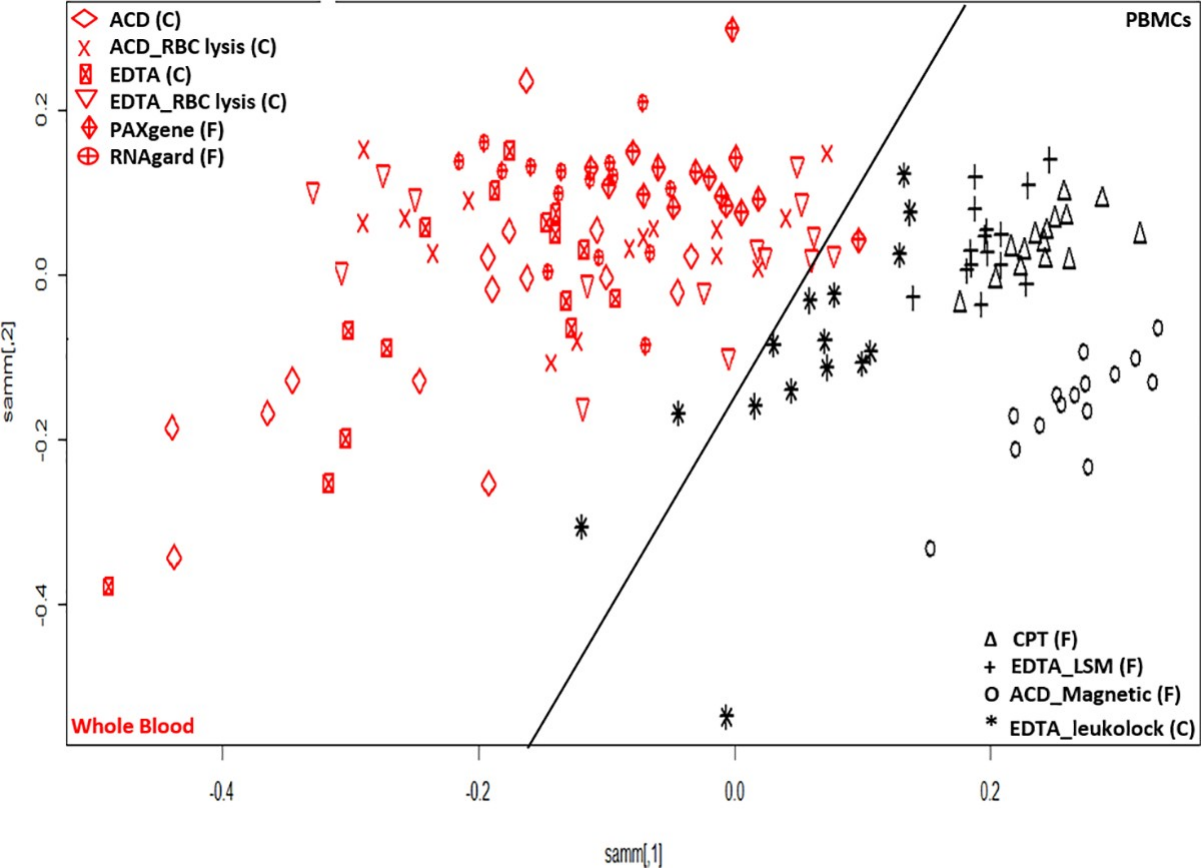
**Two dimensional Sammon projection using the top 50% of most variable transcripts (25370) for whole blood (red) and PBMC (black).** There is a clear distinction in clusters for PBMCs and whole blood sample processing as marked by a black tilted line. All whole blood samples are shown on left side and PBMC samples are on the right side of the plot. The (F) and (C) denotes the frozen and cold conditions for the sample collection and storage. The description of all the legends is given here. **PBMCs samples:** *ACD_Magnetic*- blood collected in ACD-A tubes and separated using magnetic method followed by extraction using TRIzol^®^ method. *CPT*—blood collected in CPT tubes and PBMCs separation using manufacturer recommendations followed by extraction using TRIzol^®^ method. *EDTA_LSM*- blood collected in EDTA tube and PBMC separation using LSM followed by extraction using TRIzol^®^ method. *EDTA_Leukolock*- blood collected in EDTA tube, PBMCs separated using LeukoLOCK^™^ method followed by extraction using TRIzol^®^ method. **Whole blood samples:** *ACD*- blood collected in ACD-A tube followed by RNA extraction using TRIzol^®^ LS method. *ACD_RBC lysis-* blood collected in ACD-A tube followed by RBC lysis using ACK buffer and RNA extraction using miRNAeasy extraction kit. *EDTA_RBC lysis* -blood collected in EDTA tube followed by RBC lysis using ACK buffer and RNA extraction using miRNAeasy extraction kit. *EDTA*- blood collected in EDTA tube followed by RNA extraction using TRIzol^®^ LS method. *PAXgene*- blood collected in PAXgene^®^ tube followed by RNA extraction using PAXgene^®^ blood miRNA kit. *RNAgard—*blood collected in RNAgard^®^ tube followed by RNA extraction using Biomaxi^™^ Precip Buffer and PAXgene^®^ Blood miRNA kit.

The most variable genes analysis (Fig 3) for gene expression profile of these samples using the top 50% variable genes in the dataset (25370 probes) showed a clear distinction between whole blood and PBMC samples, resulting in clusters with distinct separation of these groups. We observed that the majority of whole blood samples (EDTA as well as ACD-A tubes) when processed using ACK Lysing Buffer were grouped next to the PBMC clusters. The ACK Lysing Buffer is used to lyse erythrocytes in whole blood samples and is not known to have any impact on RNA extraction procedures [36]. Erythrocytes burst rapidly in the presence of a hypotonic buffer and thus allow fast removal of erythrocytes without affecting the stability of the leukocytes. Based on these clusters, there seems to be leukocyte selection happening for these samples leading to their clustering along with PBMC samples. In general, the complete list of all the genes with their variance score (R var function) is shown in S1 Table. The PCA generated was created by using the top 50% of variable genes (Fig 3). We did not observe any samples from the same individual grouping together, indicating the strong effects of the sample processing pipeline on the gene expression profile. We further observed a clear separation between the PBMC extraction procedures and found that magnetic separation is a distinct subgroup. The PBMC extraction procedure using CPT tubes had a good overlap with PBMCs processed using the FICOLL^™^ procedure. It has been previously shown that PBMC isolation methods for CPT tubes and density gradient methods did not impact the gene expression profile of the cells [7]. Fig 3 shows that the LeukoLOCK^™^ PBMC procedure samples grouped together and closer to the whole blood gene expression procedures. In the whole blood procedures, samples processed using ACK Lysing Buffer were clustered closer to the PBMC procedures, which relates to the RNA extraction from lymphocytes after erythrocytes depletion (Fig 3). The RNAgard^®^ and PAXgene^®^ RNA preserved samples are clustered more closely on the PCA graph.

### Effect of shipping temperature on the gene expression pattern in whole blood and PBMCs

**Fig 4:**
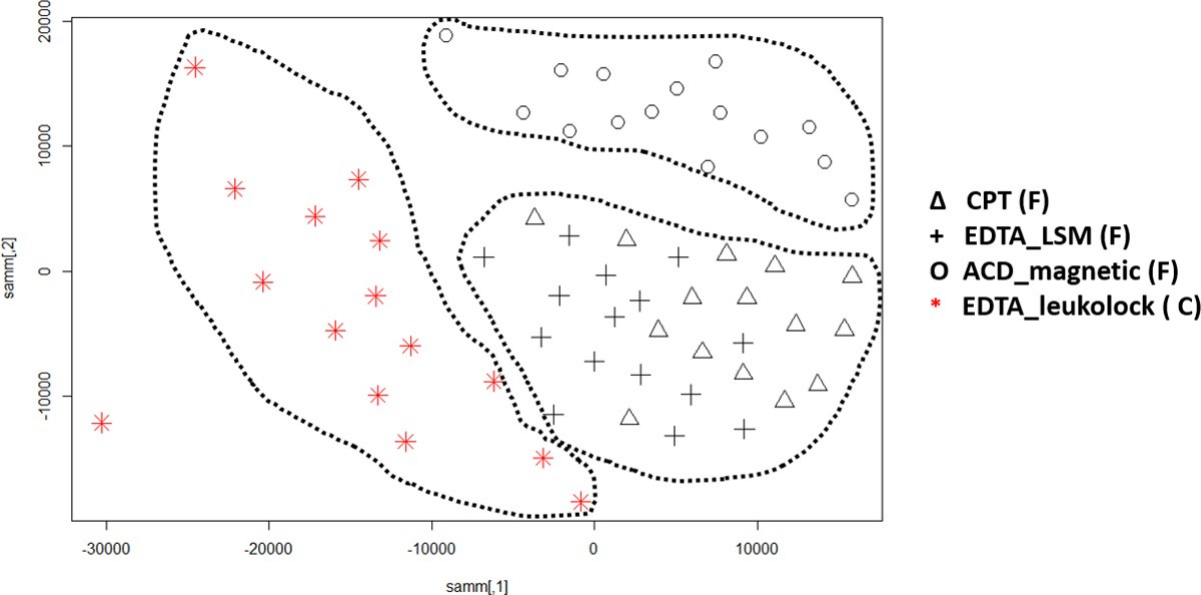
**Sammon mapping using Manhattan distance** of all probes for PBMCs sample separation methods where non-frozen LeukoLOCK^™^ separated (red) and cryopreserved samples (black) are shown as two distinct groups. The legends are described Fig 3. The CPT tubes and LSM separated PBMCs are clustered together whereas magnetic separation and LeukoLOCK^™^ separated PBMCs are clustered as distinct clusters with all clusters marked using a dotted line. The (F) and (C) denotes the frozen and cold conditions of the sample storage before RNA extraction.

It is important that the PBMC samples are processed and preserved immediately to obtain the best gene expression data. In our study, the PBMCs were isolated immediately and frozen or stabilized without freezing using the commercially available LeukoLOCK^™^ method by capturing the total leukocyte population and eliminating red blood cells, platelets, and plasma. The RNA was stabilized using RNA*later^®^* solution, which can be maintained for several days at room temperature. We preserved LeukoLOCK^™^ samples in cold conditions after receiving the shipment on ice. The data analysis of these frozen to cold shipped groups of PBMCs showed 20962 (FDR corrected at p-value < 0.05) DEGs that were differentially expressed between cold and cryopreserved samples. Of these 20,962 probes, 8,459 were downregulated in cryopreserved samples and 12,503 were upregulated in cold storage samples. The transcripts with increased signal intensity exhibited a maximal log2 fold change of 2.99 and the transcripts with decreased signal intensities exhibited log fold changes up to -4.8. A Sammon plot using the Manhattan distance (Fig 4) for all probes showed distinct patterns for LeukoLOCK^™^ samples as shown by red colored samples. The figure also shows that magnetic separation followed by cryopreservation results in a slightly different grouping than FICOLL^™^ and CPT tube sample preparation.

**Fig 5:**
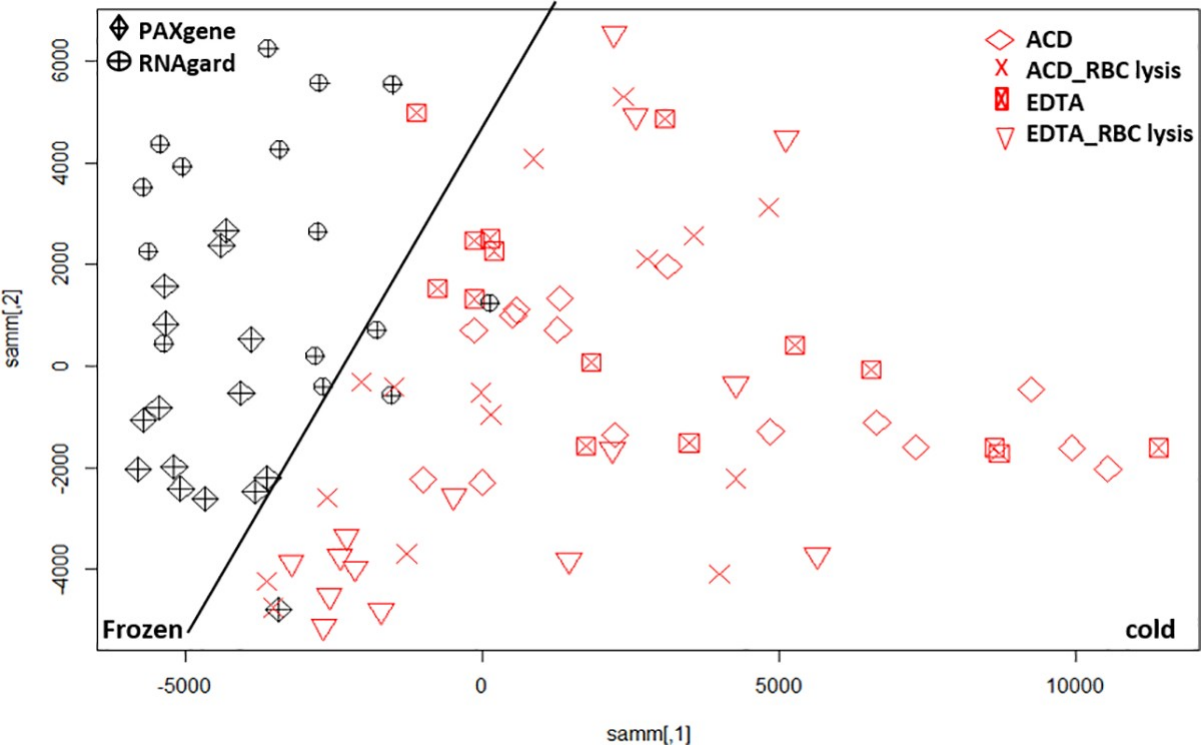
**Sammon mapping using Manhattan distance of significant probes (p<0.05) for whole blood sample preparation methods where non-preserved/cold conditions (red) and frozen/cryopreserved samples (black) are shown as two distinct groups and are shown separated by a black line.** The legends are described in Fig 3.

**Fig 6:**
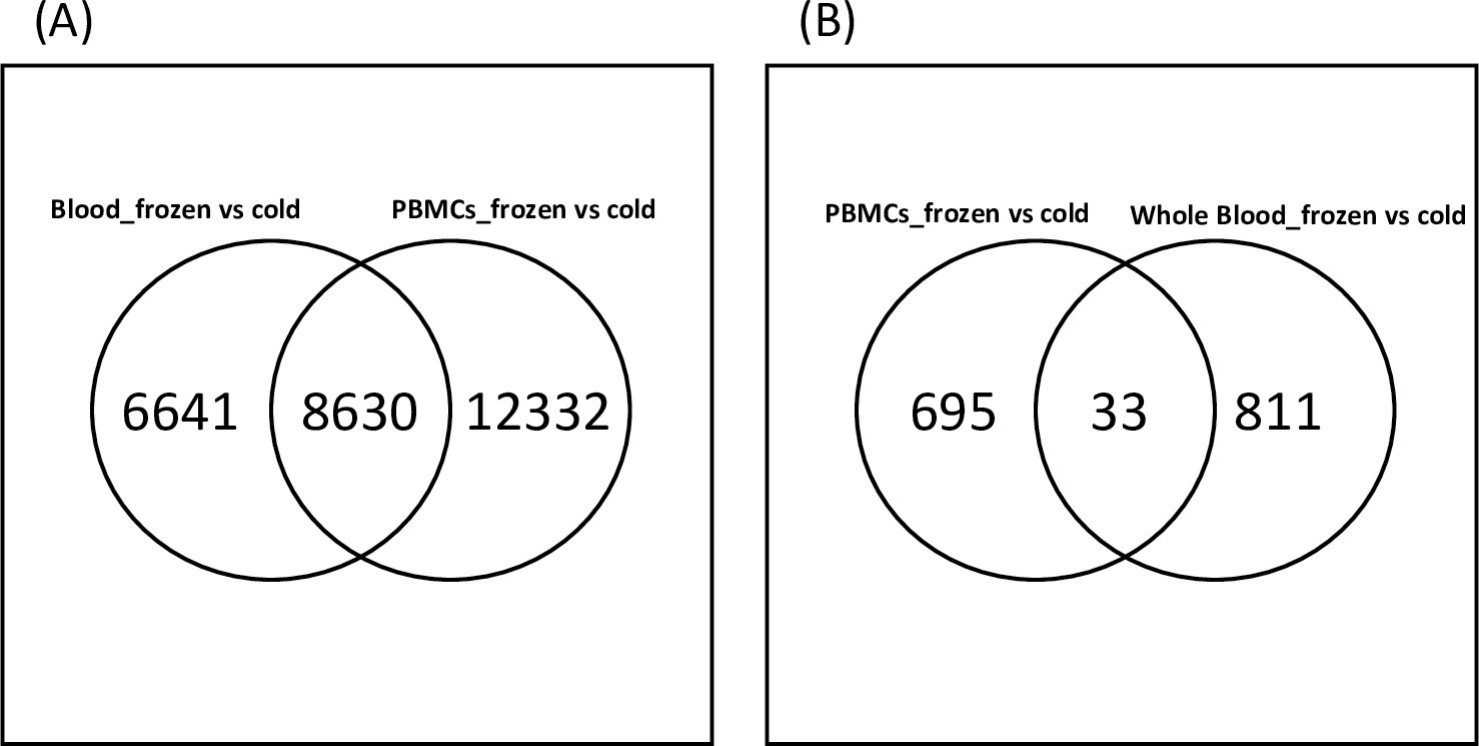
**(A)** Venn diagram showing the common number of DEGs where comparisons of PBMCs and whole blood are affected by cryopreservation (frozen) vs. cold conditions are done. **(B)** Venn diagram showing the common DEGs from top 1000 mapped IDs using IPA where comparisons of PBMCs and whole blood are affected by cryopreservation (frozen) vs. cold conditions.

The whole blood analysis used cryopreserved PAXgene^®^ Blood RNA and RNAgard^®^ samples. These products use proprietary reagents to stabilize RNA, ensuring the gene expression profiles are preserved from the moment of sampling. However, using these tubes can increase the sample processing cost, and it may not always be possible to ship and process cryopreserved samples overnight. Considering this situation, we also studied gene expression profiling of blood collected in either EDTA or ACD-A tubes followed by extraction with and without RBC lysis. We do consider that viability, processing time, and additional unknown issues may affect gene expression. The analysis of cold to cryopreserved groups of blood samples showed 15,271 (FDR corrected at p value <0.05) and 10,766 (FDR corrected at p value < 0.01) probes were differentially expressed between cold and cryopreserved samples. Of these 15,271 probes, 6,947 were downregulated in cryopreserved samples and 8,324 were upregulated in cold storage samples. The transcripts with increased signal intensities exhibited a maximal log2 fold change of 2.12 and transcripts with decreased signal intensities exhibited log2 fold changes up to -5.39. Manhattan distance analysis showed two distinct groups where majority of the cryopreserved samples were distinctly in one cluster separated from the other methods by a black bar in Fig 5. We looked at DEGs (FDR ≤ .05) across cryopreserved or cold whole blood and PBMC samples. Of all the probes affected by preservation temperature in PBMCs and whole blood, 8,630 were shared between the whole blood and PBMCs (Fig 6A) whereas 6,641 were unique to cold vs. frozen whole blood and 12,332 were unique to cold vs. frozen PBMC.

### Effect of storage temperature on the pathway analysis in blood and PBMCs

To elucidate if cryopreservation influenced biological processes, the DEG list was filtered by selecting the top 1000 FDR corrected p-values in PBMCs as well as whole blood and analyzed using IPA (S2 Table). The PBMC samples showed no canonical and no biofunctions significantly affected by this filtered gene list after multi-test correction. There were 33 DEGs common to both groups (Fig 6B).

The DEGs from the whole blood samples were enriched for multiple biofunctions after multi-test correction (Benjamini-Hochberg method). The top biological function was hematological system development and function, which had 127 functions (Table 3) in this group, of which 97 were significant at p-value <0.05. The most significantly enriched biofunctions are the quantity of different cell types, cells accumulation, cell movement, cell activation, cell recruitment, etc.

**Table 3:** The functions associated with hematological system development: Top 1000 FDR corrected DEGS from whole blood when frozen vs. cold conditions are compared using BH p-value (Benjamin-Hochberg corrected p-value) settings in IPA.

Diseases or Functions Annotation	p-Value	# Molecules
Quantity of mononuclear leukocytes	5.82E-14	85
Quantity of lymphocytes	9.93E-13	80
Quantity of blood cells	4.24E-12	102
Quantity of leukocytes	9.47E-12	93
Quantity of T lymphocytes	2.85E-09	58
Quantity of hematopoietic progenitor cells	6.46E-09	50
Quantity of B lymphocytes	7.11E-08	40
Cell movement of natural killer cells	2.80E-07	14
Morphology of lymphoid tissue	3.79E-07	53
Lymphopoiesis	4.44E-07	62
Abnormal morphology of lymphoid organ	6.91E-07	39
Cell movement of leukocytes	9.72E-07	71
Recruitment of natural killer cells	9.82E-07	7
Cell movement of lymphocytes	1.09E-06	42
Function of T lymphocytes	1.18E-06	29
Accumulation of blood cells	1.20E-06	32
Formation of lymphoid tissue	1.33E-06	37
Morphology of lymphoid organ	1.53E-06	48
Quantity of thymocytes	1.55E-06	27
Differentiation of mononuclear leukocytes	1.98E-06	63
Leukopoiesis	3.67E-06	69
Cell movement of mononuclear leukocytes	4.43E-06	46
Homing of lymphocytes	4.63E-06	20
NK cell migration	4.69E-06	10
Differentiation of B lymphocytes	5.52E-06	26
Accumulation of leukocytes	6.14E-06	29
Extravasation of phagocytes	7.08E-06	8
Migration of mononuclear leukocytes	7.10E-06	38
Lymphocyte migration	7.32E-06	36
Function of lymphocytes	7.55E-06	31
Chemotaxis of lymphocytes	1.08E-05	17
Morphology of spleen	1.18E-05	37
Abnormal morphology of thymus gland	1.22E-05	18
Quantity of monocytes	1.23E-05	16
Homing of leukocytes	1.35E-05	37
Abnormal morphology of germinal center	1.39E-05	11
Activation of lymphocytes	1.56E-05	41
Recruitment of T lymphocytes	1.84E-05	12
Chemotaxis of leukocytes	2.25E-05	35
Activation of mononuclear leukocytes	2.43E-05	42
T cell homeostasis	2.61E-05	48
Recruitment of lymphocytes	2.83E-05	14
Extravasation of leukocytes	3.45E-05	9
Formation of lymphoid organ	3.81E-05	28
Proliferation of mononuclear leukocytes	4.44E-05	60
Accumulation of mononuclear leukocytes	4.89E-05	18
Morphology of thymus gland	5.17E-05	21
Proliferation of lymphocytes	5.54E-05	59
Extravasation of neutrophils	6.73E-05	6
Activation of blood cells	6.92E-05	58
Formation of lymph node	7.62E-05	14
Proliferation of immune cells	7.95E-05	61
Function of granulocytes	8.19E-05	8
Homing of mononuclear leukocytes	8.40E-05	22
Chemotaxis of natural killer cells	8.60E-05	7
Quantity of regulatory T lymphocytes	9.13E-05	15
Abnormal morphology of spleen	9.44E-05	26
Cell movement of T lymphocytes	9.67E-05	24
T cell migration	1.21E-04	26
T cell development	1.25E-04	45
Recruitment of leukocytes	1.45E-04	30
Development of hematopoietic system	1.51E-04	30
Morphology of lymph follicle	1.62E-04	15
Accumulation of lymphocytes	1.63E-04	16
Quantity of short-term hematopoietic stem cells	1.73E-04	3
Delay in accumulation of leukocytes	1.73E-04	3
Function of myeloid cells	2.03E-04	20
Recruitment of mononuclear leukocytes	2.37E-04	15
Activation of CD8+ T lymphocyte	2.45E-04	6
Chemotaxis of mononuclear leukocytes	2.65E-04	19
Emigration of mononuclear leukocytes	2.79E-04	4
Abnormal morphology of enlarged spleen	3.31E-04	17
Activation of T lymphocytes	3.43E-04	30
Cell proliferation of T lymphocytes	3.47E-04	47
Activation of leukocytes	3.65E-04	52
Quantity of CD4+ T-lymphocytes	3.99E-04	17
Migration of follicular B lymphocytes	4.26E-04	4
Egression of leukocytes	4.27E-04	5
Maturation of leukocytes	4.35E-04	21
Cell movement of B lymphocytes	5.33E-04	12
Development of bone marrow	6.03E-04	20
Accumulation of myeloid cells	6.15E-04	18
Quantity of long-term hematopoietic stem cells	6.20E-04	4
Accumulation of blood platelets	6.20E-04	4
Formation of thymus gland	6.82E-04	17
Response of lymphocytes	7.17E-04	19
Differentiation of T lymphocytes	7.98E-04	32
Osteoclastogenesis of bone marrow	8.40E-04	7
Egression of lymphocytes	1.19E-03	4
Cell movement of marginal-zone B lymphocytes	1.19E-03	4
Proliferation of B lymphocytes	1.24E-03	25
Circulation of blood	1.32E-03	9
Accumulation of phagocytes	1.39E-03	15
Deposition of blood platelets	1.40E-03	3
Differentiation of colony-forming erythroid cells	1.40E-03	3
Size of lymphoid organ	1.44E-03	10
Cell movement of cytotoxic T cells	1.57E-03	4
Abnormal quantity of leukocytes	1.99E-03	10
Homing of T lymphocytes	2.02E-03	12
Differentiation of B-1 lymphocytes	2.04E-03	4
Formation of germinal center	2.05E-03	8
Osteoclastogenesis of leukocytes	2.05E-03	7
Trafficking of macrophages	2.18E-03	3
Recruitment of blood platelets	2.18E-03	3
Activation of cytotoxic T cells	2.30E-03	7
Formation of spleen	2.33E-03	10
Homing of B lymphocytes	2.35E-03	6
Binding of professional phagocytic cells	2.38E-03	18
Quantity of myeloid cells	2.57E-03	37
Development of monocyte-derived macrophages	2.59E-03	4
Osteoclastogenesis of macrophages	2.68E-03	6
Aggregation of blood platelets	2.72E-03	18
Expansion of lymphocytes	2.87E-03	16
T cell response	3.12E-03	15
Expansion of leukocytes	3.14E-03	17
Lack of white pulp	3.19E-03	3
Proliferation of plasma cells	3.19E-03	3
Accumulation of granulocytes	3.19E-03	11
Activation of naive T lymphocytes	3.24E-03	4
Migration of thymocytes	3.24E-03	4
Osteoclastogenesis of bone marrow-derived macrophages	3.25E-03	5
Quantity of double-positive thymocyte	3.41E-03	11
Recruitment of macrophages	3.41E-03	11
Maturation of antigen presenting cells	3.55E-03	12
Quantity of follicular B lymphocytes	3.55E-03	12
Morphology of lymph node	3.62E-03	15
Delay in accumulation of T lymphocytes	3.69E-03	2

It is normally recommended that hematological analysis needs be performed immediately after sample collection or at the most within 24 hours [37]. Gene expression of some selected genes also seemed to be influenced by transport and storage of blood samples at 4°C without any stabilizing solution [38]. It has been shown that storage temperature affected RBC aggregation and storage at cold conditions lead to stable aggregation for 12 hours [39]. In our study, we observed that storing and shipping human blood samples cold overnight definitely had an impact on the hematological system, as demonstrated by the DEGs for cold vs. cryopreserved whole blood and PBMC samples. Additionally, storage temperature effects the quality of downstream processing, and recently it has been shown that storage at room temperature for less than 24 hours was critical for high-quality RNA samples for next generation sequencing, whereas microarrays were still of acceptable quality after less than 32 hours of storage at room temperature [40]. Also, an earlier study reported that high temperature affects the phagocytic activity and viability of human blood mononuclear cells [41]. However, storage at low temperatures does not keep RNA samples from degradation and storing whole blood samples in the freezer dramatically damages RNA samples [40].

**Fig 7:**
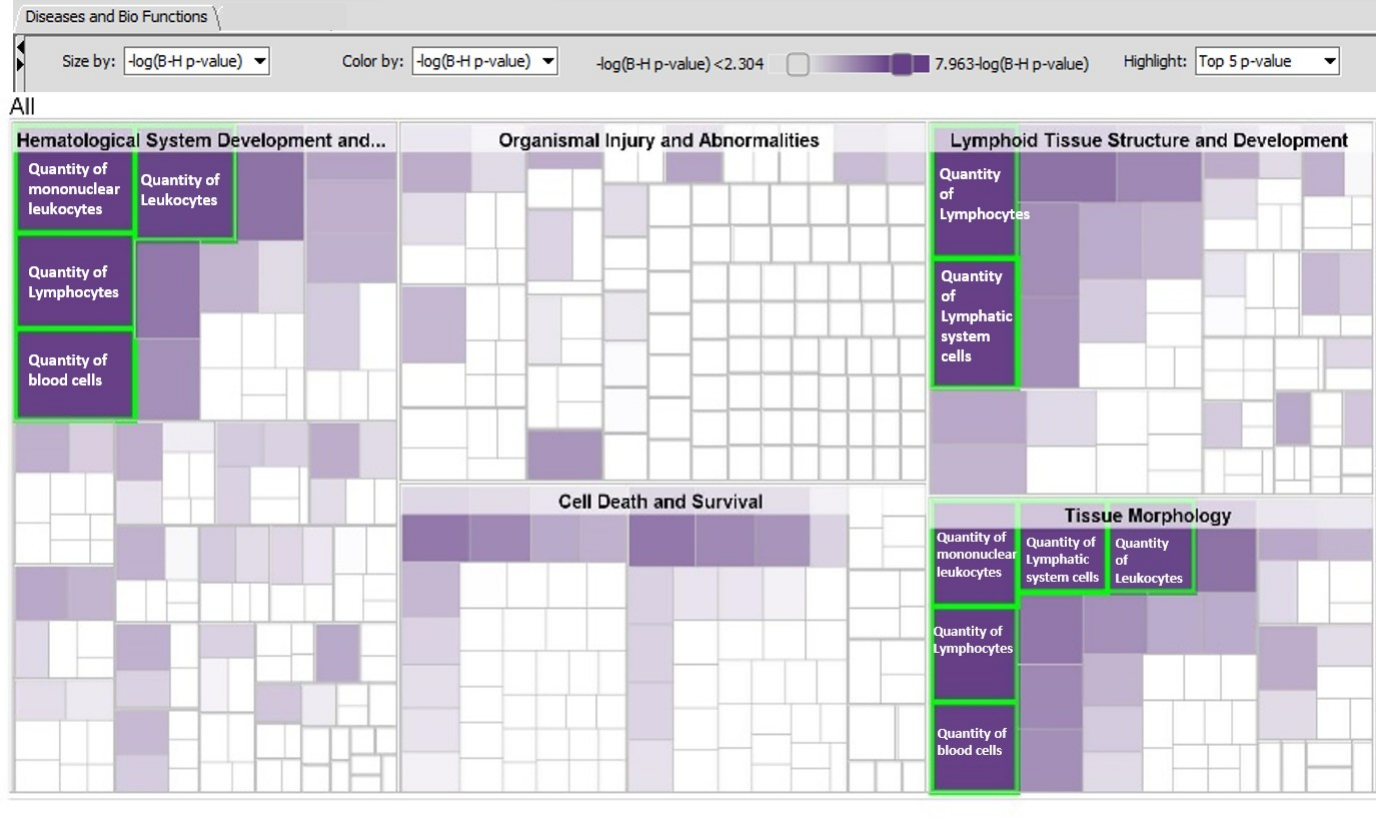
Hierarchical heatmap with diseases and bio-function category and top 5 high level functions are displayed and labeled inside the highlighted green border. The visualization is a TreeMap (hierarchical heatmap) where the major boxes represent a family (or category) of related functions. Each individual colored rectangle is a particular biological function or disease and the color indicates associated log of the calculated BH corrected p-value: lower p value (purple), or higher p value (white). Darker colors indicate lower p values. In this default view, larger squares indicate more significant overlap between the genes perturbed in the dataset and the function or disease. The image has been cropped for better readability. Here data from whole blood when frozen vs. cold conditions are compared using top 1000 FDR corrected DEGs was used.

Besides hematological system development, the other top significant biofunctions (Table 4) were related to lymphoid tissue structure and development, tissue morphology, cellular function and maintenance, and cell death and survival. For each of these categories, the number of molecules as well as the numbers of biofunctions affected are shown in Table 4. On further examination, we found that the top 5 significant biofunctions are concentrated into the functional categories of quantity of lymphocytes, leukocytes, blood cells, mononuclear cells and lymphatic system cells (Fig 7). In this study, we observed that the storage temperature of samples could confound interpretation of RNA transcript analysis and underscores its importance while planning an experiment.

**Table 4:** Biofunctions and diseases in the order of p-value affected in whole blood when frozen vs. cold conditions are compared using top 1000 FDR corrected DEGs. BH p-value refers to the Benjamini-Hochberg corrected p-value in IPA.

Category	B-H p-value	# of molecules	# of functions
Hematological System Development and Function	6.68E-10-8.11E-02	167	127
Tissue Morphology	6.68E-10-8.11E-02	149	45
Lymphoid Tissue Structure and Development	5.71E-09-8.11E-02	128	73
Cellular Function and Maintenance	4.68E-06-3.06E-02	81	13
Cell Death and Survival	7.95E-06-8.11E-02	233	87
Hematopoiesis	7.95E-06-8.01E-02	91	21
Organ Morphology	4.33E-05-8.11E-02	86	23
Humoral Immune Response	5.1E-05-8.11E-02	65	20
Cancer	7.21E-05-7.78E-02	209	42
Hematological Disease	7.21E-05-8.11E-02	172	46
Immunological Disease	7.21E-05-7.96E-02	199	52
Organismal Injury and Abnormalities	7.21E-05-8.01E-02	313	115
Cellular Movement	1.69E-04-8.01E-02	134	51
Immune Cell Trafficking	1.69E-04-8.11E-02	96	49
Inflammatory Disease	1.7E-04-7.96E-02	125	15
Inflammatory Response	1.7E-04-8.11E-02	185	49
Neurological Disease	1.7E-04-8.01E-02	128	22
Cellular Development	2.22E-04-8.11E-02	172	37
Cellular Growth and Proliferation	2.22E-04-7.78E-02	161	32
Embryonic Development	2.22E-04-7.78E-02	144	21
Organ Development	2.22E-04-7.73E-02	121	21
Organismal Development	2.22E-04-7.73E-02	162	36
Tissue Development	2.22E-04-8.11E-02	143	50
Cellular Assembly and Organization	2.22E-04-7.73E-02	19	4
DNA Replication, Recombination, and Repair	2.22E-04-7.72E-02	33	7
Post-Translational Modification	2.22E-04-3E-02	64	4
Protein Synthesis	2.22E-04-8.11E-02	136	14
Protein Trafficking	2.22E-04-7.17E-02	46	4
Cell Cycle	2.91E-04-8.11E-02	127	15
Gene Expression	2.91E-04-8.11E-02	161	14
Cell-To-Cell Signaling and Interaction	3.22E-04-8.01E-02	97	30
Cell-mediated Immune Response	3.22E-04-7.77E-02	67	13
Cell Morphology	1.03E-03-8.11E-02	57	16
Infectious Diseases	1.09E-03-6.22E-02	134	8
Connective Tissue Disorders	2.72E-03-7.96E-02	102	14
Skeletal and Muscular Disorders	2.72E-03-7.96E-02	152	16
Cardiovascular System Development and Function	3.16E-03-7.73E-02	95	16
Cardiovascular Disease	4.49E-03-7.73E-02	52	11
Developmental Disorder	4.49E-03-6.33E-02	42	10
Cell Signaling	6.96E-03-4.26E-02	47	32
Hereditary Disorder	7.19E-03-6.33E-02	30	6
Free Radical Scavenging	7.76E-03-1.67E-02	42	2
Renal and Urological Disease	7.96E-03-7.77E-02	95	6
Dermatological Diseases and Conditions	8.1E-03-5.59E-02	64	4
Gastrointestinal Disease	8.51E-03-7.73E-02	58	10
Hepatic System Disease	8.51E-03-7.73E-02	44	8
Antimicrobial Response	8.51E-03-1.67E-02	28	2
Connective Tissue Development and Function	1.48E-02-7.78E-02	69	11
Psychological Disorders	2.06E-02-3.5E-02	59	2
Nervous System Development and Function	2.38E-02-4.1E-02	7	3
Visual System Development and Function	2.38E-02-8.11E-02	11	2
Skeletal and Muscular System Development and Function	2.45E-02-8.11E-02	67	14
Cellular Compromise	2.9E-02-8.02E-02	36	7
Metabolic Disease	3.05E-02-6.33E-02	93	3
Ophthalmic Disease	4.1E-02-4.1E-02	2	1
RNA Post-Transcriptional Modification	4.1E-02-4.1E-02	2	1
Renal and Urological System Development and Function	4.1E-02-6.7E-02	16	2
Molecular Transport	4.26E-02-7.17E-02	57	4
Vitamin and Mineral Metabolism	4.26E-02-4.26E-02	21	1
Digestive System Development and Function	4.4E-02-7.73E-02	27	2
Hepatic System Development and Function	4.4E-02-7.73E-02	27	2
Tumor Morphology	5.1E-02-7.73E-02	32	2
Reproductive System Disease	6E-02-6.09E-02	44	2
Endocrine System Disorders	6.09E-02-6.09E-02	3	1
Reproductive System Development and Function	6.09E-02-6.09E-02	3	1
Organismal Survival	6.32E-02-6.32E-02	155	1
Energy Production	6.7E-02-6.7E-02	4	1
Nucleic Acid Metabolism	6.7E-02-6.7E-02	4	1
Small Molecule Biochemistry	6.7E-02-6.7E-02	4	1
Respiratory Disease	6.9E-02-7.73E-02	9	3

## Conclusions

Blood cells are suitable for gene expression analysis but methods of collection, storage, and extraction may affect transcription profiles. Here, we studied each of these factors and expanded our knowledge regarding the variation in gene expression from blood samples collected from healthy participants. Our results provide new insights into RNA isolation from blood as well as from PBMC samples, revealed how the choice of storage, handling, and extraction methodologies influences RNA isolation quality and apparent gene expression, and how careful consideration is necessary to avoid bias resulting from downstream processing. Better characterization of the effects of collection method idiosyncrasies will facilitate further research into understanding the effect of gene expression on variability in human sample types. These results point out the need for a strict standardization of handling the blood specimen with regards to peripheral blood sample processing time between phlebotomy and RNA isolation.

## Supporting information

S1 Table List of high variance probes. (XLSX)

S2 Table Top 1000 DEGs in PBMC when frozen conditions are compared to cold conditions. (XLSX)

S3 Table Top 1000 DEGs in whole blood when frozen conditions are compared to cold conditions. (XLSX)
